# Topics of Public Interest

**Published:** 1915-07

**Authors:** 


### TOPICS OF PUBLIC INTEREST.

---

**Narcotic Law Ruling**—State vs. Norwood Supreme Court, N. J. March 23, 1915. Reversing a conviction made in the lower court under the following statute:

> "Any person who shall sell, give away furnish or dispose of the alkaloid cocaine, or its salt, alpha or beta eucaine or their salts, opium, morphine or their salts, opium morphine, codine chloral or any of the derivatives of chloral, or who shall sell or give away, furnish or dispose of any admixture of cocaine or eucaine or any patent or proprietary remedy containing cocaine or eucaine except on the written prescription of a duly licensed and practicing physician, shall be guilty of a misdemeanor."

the court bases its action on the doctrine that the statute being penal in its object the rules of statutory construction must apply; that is, interpretation cannot be enlarged by judicial construction.

It appeared that the drug clerk of the defendant sold a bottle containing tablets each tablet containing 1-12 of a grain of heroin. Witnesses for the state testified that heroin was in fact morphine, while the defendants' contended that these were two different drugs, and that the effects were different in different persons. The court ruled that heroin was not included in the catagory of inhibited habit-forming drugs as included in the above statute, that if the legislature knew of the drug at the time of the enactment it was purposely excluded, and if it was not known manifestly the legislature could not have had it in mind.

The court also took into consideration the fact that the storekeeper himself did not commit the offense, that the clerk was unaware of the drug coming under the head of or category of habit forming drugs and as soon as he learned of the mistake, the sale was discontinued. On these grounds the conviction of the lower court was reversed.

Frank Engers, 129 S. Bond Street, Baltimore, Md.

---

**Medical and Ethical Problems of the War.** The occurrence of a dozen cases of insanity among German sailors on merchant ships lying in New York harbor has been variously explained as due to idleness, other strictly psychic factors and to insufficient and spoiled food. Investigation by the Health Dept. has shown that the food served on different ships differs. In some instances, the food seemed to be excellent—particularly the war bread—and abundant, in others, some excuse for complaint was found.

The use of toxic gases—apparently mixtures of chlorin and bromin—in warfare is a revival of ancient practices on a scientific basis. We would advocate a strictly neutral attitude, both in the political sense and as regards the humanitarian conduct of war viewed from the medical standpoint. It is altogether likely that the practical application of well-known chemic and physical principles will provide means of defense relatively as perfect as against missiles. For instance, a stream of air or water of great force would be as feasible as a powerful gun and respirators with chemic neutralization have already been introduced. War necessarily involves killing and it cannot be reduced to vivisectional euthanasia. Personally, we would as lief be choked to death as punctured in a vital organ. Recent press reports doubtless exaggerate the cruelty of the pulmonary effects or irrespirable gases. Efficiency in the sense of producing as nearly as possible a 100 per cent. death rate from a given attack is the object of all kinds of military offensive measures. Practically all are agreed that the present war must be fought to a decisive finish and this means the destruction or disablement, relatively permanent, of a critical percentage of the fighting force of one side. The more quickly this can be accomplished, the more humane will the war be in its ultimate effects.

The problems of reprisals, of rape and its after-conduct, are sad ones. Let it be emphasized that these problems depend on human rather than on minor racial tendencies.

The medical profession is vitally interested in many problems of war, both in regard to military medical service and to general scientific and therapeutic advance. It is also a large factor in the molding of public opinion. We urge a concentration of attention on these matters rather than a yielding to passion and the subordination of native and hereditary sympathies to national duty at a time when it is most important that this country should act as a unit and most deliberately, unswayed by excitement. The American profession should also exert a strong influence to maintain the world-wide esprit de corps and harmony of medical men in their professional interests and personal relations, at a time when there seems to be so marked a tendency to override this professional unity by prejudices of a political nature.

---

**Railroad Accidents.** The Chicago & North Western R. R. gives the following statistics: In the 4 1-2 years ending December 31, 1914, as compared with the 4 1-2 years ending June 30, 1910, 173 fewer employees killed, 35.3 per cent. decrease; 10,671 fewer employees injured, 27.3 per cent. decrease; 961 fewer passengers killed, 22.8 per cent. decrease; 210 fewer outsiders killed, 19.4 per cent. decrease; 228 fewer outsiders injured, 8.2 per cent. decrease; the mileage having increased from 7,953 at the end of the first period to 8,423 at the end of the second. Other statistics illustrated by circles divided into sectors, are perhaps even more instructive: For example, the collective statistics of three years showed that 185 employes (91.6 per cent.) were killed in little accidents whose prevention would have required less time than even making the required report as compared with 17 (8.4 per cent.) in major accidents including derailments and collisions. The differences for injuries were, of course, even greater, 17, 380 (97.8 per cent.) as compared with 401 (2.2 per cent.). Thoughtlessness and carelessness were responsible for 106 or 80.3 per cent. of deaths of employees, derailments and collisions for 14 or 10.6 per cent.; defects in engines, cars, tracks, tools, etc., for 11 or 8.14 per cent.; and only 1 death or 0.76 per cent. was considered strictly unavoidable. It should be noted that these statistics are compiled not only as a matter of scientific interest but are graphically stated and distributed among employees to prevent further disasters.

---

**Cultivation of Drug Plants.** Farmers' Bulletin No. 663 of the U. S. Department of Agriculture, advises that there is no enormous profit in crude drugs and that the methods of propagation and harvesting and cultivation, also conditions of demand and supply, must be carefully studied. Under proper climatic and telluric conditions, the following are suggested:

Anise, Belladonna, Burdock, Caraway, Catnip, Camomile, Conium, Coriander, Digitalis, Dill, Echinacea, Elecampane, Fennel, Henbane, Horehound, Pennyroyal, Sage, Stammonium, Tansy, and Thyme.

---

**Examination of Dentists for U. S. Army.** Candidates will be examined at various places Monday, October 18. Acting Dental Surgeons are employed on a 3-year contract, at $150 a month with certain perquisites. If found qualified at the expiration of the contract, they are permanently appointed Dental Surgeons, with rank of first lieutenant and pay and allowances of that rank. Application blanks and full information may be obtained of the Surgeon General, U. S. A., Washington, D. C.

---

**Automobile Caution.** The police regulation for Buffalo has been changed back to a prohibition of leaving machines on Main Street between Seneca and Chippewa, from 8 a. m. till 7 p. m. On Washington and Pearl Streets the hour limit prevails. Machines may stop on Main Street to discharge and take on passengers and may stand a few minutes if in charge of someone who can start and manage them. Dogs and persons not competent and authorized to act as chauffeurs do not count as attendants. In touring, we would advise strict obedience to general regulations, inquiry of the first policeman in any city, as to peculiar local regulations and prompt and unquestioning obedience to direct orders. In some places, certain crossings are restricted to "straight traffic" (no right-angled turn allowed) and in others traffic is diverted from certain parts of diagonal streets on to cross streets. Inexplicable objections by the police to apparently normal courses may be due to temporary obstructions.

---

**Limitation of Offspring** is beginning to be discussed favorably by sociologists. We trust that the obvious and very trite arguments will not occupy too much space in literature. However, it will do no harm to have the matter threshed out from the ethical and economic standpoints. It should be understood that nothing can be accomplished on a large scale till laws regarding not only the sale of preventatives but the publication of methods have been repealed, and it is just as well that this conservative factor is present. Some of our contemporaries are fairly bursting with eagerness to tell what they know in this matter.

---

**Anti-Narcotic Legislation.** The Erie County Pharmaceutical Association has adopted a resolution against filling prescriptions for narcotics till the present legal snarl has been unraveled. Perhaps it is just as well that narcotics should be administered or dispensed, a few doses at a time by physicians.

**Comparative Temperatures of Thermal Springs:**

| Places. | Name of Springs. | Tem. Deg. F. |
|---|---|---|
| California | Arrowhead | 202 |
| Carlsbad (Bohemia) | Sprudel | 164 |
| Weisbaden (Germany) | Kochbrunnen | 156 |
| Baden Baden (Germany) | Hauptquelle | 155 |
| Amelie-les-Baines (Pyrenees) | Fountaine Arago | 145 |
| Carlsbad (Bohemia) | Theresienbrunnen | 131 |
| Aix-le-Chappelle (Germany) | Kaiserquelle | 131 |
| Leuk (Switzerland) | Hauptquelle | 125 |
| Arkansas (U. S. A.) | Arkansas | 122 |
| Gastein (Austria) | Hauptquelle | 120 |
| Teplitz (Bohemia) | Hauptquelle | 120 |
| Bath (England) | King's Bath | 119 |
| Aix-les-Bains (Savoy) | Alum Spring | 116 |
| Carlsbad (Bohemia) | Schlossbrunnen | 113 |
| Bareges (Pyrenees) | Le Tambour | 113 |
| Gastein (Austria) | Doctorsquelle | 111 |
| Vichy (France) | Grand Grille | 108 |

**Health Nurse for Batavia.** The Common Council and the Board of Education have agreed to share the expense of a nurse for poor patients and for inspecting school children.

**Visiting Tuberculosis Nurses** in the proportion of 1 to 100 reported cases have been requested in Los Angeles. The measure originated with Dr. George E. Malsbury, Editor of the Southern California Practitioner and has been presented to the City Council on petition of the electors.

**The Advantages of Medical Associations** have been well presented to the Tri-State Medical Association of the Carolinas and Virginia by the President, Dr. Edward C. Register, Editor of the Charlotte Medical Journal.

**Child Welfare Day** was observed Sunday, June 20, at the instigation of Health Commissioner Biggs who, by the way, is promised the support of Gov. Whitman, irrespective of the requirement of whole time to the duties of the office. Clergymen generally were requested to deliver sermons on the conservation of children. The educational campaign of the Division of Child Hygiene of the State Department of Health, last year, made exhibits in 45 cities and provided 150 popular lectures, besides exhibits at county fairs, etc. This campaign "brought about a decrease in the infant death rate from 137

to 112 per 1,000 births"—saving approximately 1,400 infants' lives. (Note: Following the custom of private medical reports, one would say that the death rate was 112 as contrasted with a previous rate of 137. It is not entirely certain that the 1914 death rate will not be exceeded in future years nor that the diminution was entirely due to the campaign mentioned, excellent as it is.)

---

**Harmless X-Hay.** C. H. Stanley of New York, claims to have perfected a modification which will render it possible to operate under the X-Ray or to employ long exposures without danger to patient or attendant. Details are not available.

---

**Cancer in New England.** In 1913, there were 49,928 deaths from cancer in the U. S. Registration Area—78.9 per 100,000. The rates for New England were: Connecticut 85.1; Rhode Island, 93.3; Massachusetts, 101.4; New Hampshire, 104.4; Maine, 107.5; Vermont, 111.7. The high rates are probably due to comparatively small immigration and other causes of a relatively high average age of population. Popular educational campaigns have been instituted by Massachusetts and Vermont, to lead to early diagnosis and treatment.

---

**Diminished Mortality in Pennsylvania.** Dr. Samuel G. Dixon has recently been appointed to his fourth term as Commissioner of Health, having already served ten years. The department now has over 3,000 employees. 115 tuberculosis dispensaries and 3 great state sanatoria have been established, and this disease has fallen from first to second place. Typhoid mortality has been reduced to less than a quarter. For 1914, the general mortality was 13.9 per 1,000, the lowest in history. Compared with the rates for 1906, this represents a saving of life as follows: Typhoid 18,865; tuberculosis, 11,924; diphtheria, 4,648; Pertussis, 4,091; total for these four, 40,528; total for all causes, 78,916.

---

**Warning.** Wm. Wood & Co., state that the "National Educational Association" of 130 Nassau Street, is not authorized to collect money or solicit subscriptions.

---

**A Two-Year College Course** will be required by New Hampshire for matriculants expecting to graduate in the class of 1919 or later.

---

**Harrison Law Ruling.** We understand that compliance with the provisions of this law will be accepted as a substitute for those of the Boylan Law in the State of New York.

**A Single State Hospital Commissioner,** with a salary of $10,000 has been substituted for the present commission, by the Sage bill.

---

**An Obstetric Dispensary** was opened in April in Buffalo, under the management of the Bureau of Hygiene of the municipal health department.

---

**The Abbott Alkaloidal Co.**, of Chicago, has changed its name to **The Abbott Laboratories.**

---

**A Measles Epidemic** aggregating over a thousand cases prevails in and about Buffalo.

